# Weekend warrior physical activity pattern and common mental disorder: a population wide study of 108,011 British adults

**DOI:** 10.1186/s12966-017-0549-0

**Published:** 2017-07-14

**Authors:** Mark Hamer, Stuart J. H. Biddle, Emmanuel Stamatakis

**Affiliations:** 1 School of Sport, Exercise and Health Sciences, Loughborough University, Loughborough, Leics LE11 3TU UK; 2 Institute for Resilient Regions, University of Southern Queensland, Queensland, Australia; 3 Charles Perkins Centre, Prevention Research Collaboration, School of Public Health, University of Sydney, Sydney, Australia

**Keywords:** Physical activity, Mental health, Depression, Epidemiology

## Abstract

**Background:**

The dose-response association between physical activity (PA) and mental health is poorly described. We explored cross-sectional associations between physical activity and common mental disorder (psychological distress) in ‘weekend warriors’ who do all their exercise in one or two sessions per week.

**Methods:**

Adult participants (*n* = 108,011, age = 47 ± 17 yrs., 46.5% men) were recruited from general population household-based surveys (Health Survey for England and Scottish Health Survey) from 1994 to 2004. Data were pooled and analyzed using logistic regression models. Moderate to vigorous physical activity (MVPA) was self-reported and psychological distress was measured using the 12 item General Health Questionnaire (GHQ-12).

**Results:**

Psychological distress (GHQ-12 > 3) was prevalent in 14.5% of the sample. In healthy participants an inverse association between PA and psychological distress was optimal at the PA guideline (150 mins/wk. MVPA or 75 min/wk. Vigorous PA) regardless of whether it was accumulated in one or two bouts per week “Weekend warrior” (odd ratio = 0.68, 95% CI, 0.63, 0.73) or as more frequent daily bouts (odd ratio = 0.68, 95% CI, 0.64, 0.72) in comparison to the inactive reference group. In participants with chronic health conditions an inverse association between PA and psychological distress was also evident at lower doses (one or two sessions of PA a week below PA guideline) (OR = 0.72, 95% CI, 0.68, 0.77). Undertaking vigorous intensity PA as part of the PA guideline conferred additional benefit in women (odds ratio = 0.87, 95% CI, 0.75, 1.00), but not men.

**Conclusion:**

Mental health benefits may be accrued through different PA patterns, thus individual approaches to prescribing exercise should be promoted.

**Electronic supplementary material:**

The online version of this article (doi:10.1186/s12966-017-0549-0) contains supplementary material, which is available to authorized users.

## Background

The term common mental disorders (CMDs) is used to define different types of depression and anxiety that do not meet criteria for major psychiatric diagnosis or in some cases remain undiagnosed [1]. CMDs can cause significant emotional distress and impact on daily function leading to physical, social and occupational disability and premature mortality [2]. Although usually less disabling than major psychiatric disorders, CMDs are more highly prevalent (one in six adults in England [1]), thus are likely to have higher impact to society. Over the past few decades substantial evidence has accumulated on the mental health benefits of physical activity (PA) [3–6]. Information on the dose-response association is important to inform public health guidelines and personalized approaches to exercise prescription, although existing data are equivocal. Few studies have focused on the mental health benefits of meeting the current PA guideline. The 2011 UK guidelines recommend 150 min/wk. of moderate PA, or 75 min/wk. of vigorous PA, or a combination of the two, as well as at least 2 days of muscle strength exercise per week [7]. These were developed as a ‘best fit’ for health, notwithstanding that different health outcomes may require different amounts of activity.

Further investigation is required into the mental health effects of different PA patterns, particulary with regards to frequency. While it is recommended to accumulate PA over most days of the week, results of existing observational studies suggests that compared to inactive individuals, participants may gain substantial mental health benefit by participating in PA below the current 150 min/wk. threshold, and in some studies the associations are comparable to those seen in participants meeting the full PA guidelines [8–12]. For example, being active one to two times/week, popularly phrased as the “Weekend Warrior” activity pattern [13], was associated with a decreased risk of depression of up to 40% [14, 15], although these findings have not been consistently reproduced [16].

Several reasons might explain the heterogeneity in results. Firstly, exposure measures of PA have been crude in some studies, making it difficult to fully characterize the dose-response association. Secondly, studies have investigated a wide variety of psychological outcome measures, and not all would be expected to be affected by PA in the same way. Thirdly, prior studies have often been of limited sample size making it difficult to stratify participants according to important characteristics such as sex, age, and presence of chronic disease risk factors. Evidence on dose-response associations between PA and mental health in particular groups of individuals thus remains largely unknown. Moreover, it has long been argued that dose-response outcomes for PA are too complex to consider in simple stimulus-response terms [17]. Individuals will have preferences, characteristics and activity histories that might lead to different psychological responses to similar types or loads of PA [18]. For these reasons, it is important to explore dose-response associations across different groups of people.

In a large population sample of adults we aimed to explore if mental health benefits can be optimized by accumulating PA in certain patterns (e.g., 1–2 sessions per week vs. 5–6 sessions per week; moderate vs. vigorous PA). Certain variables are known to be associated with CMDs, including sex, age, presence of chronic illness, and risk factors (eg, smoking and obesity) [1], thus we explored if associations between physical activity and CMDs were modified by these factors.

## Methods

### Participants

Adult participants were recruited from the Health Survey for England (HSE) and the Scottish Health Survey (SHS), that are cross-sectional surveys, each year containing different participants [19, 20]. Briefly, HSE and SHS are household-based surveillance studies using a multistage, stratified probability design in order to target a representative sample of the population. Stratification was based on geographical areas, not individual characteristics: postcode (zip code) sectors were selected at the first stage and household addresses selected at the second stage. Participants in the present study were derived from surveys in 1994 (HSE only), 1995 (SHS only), 1998, 1999 (HSE only), 2003, and 2004 (HSE only). Local research ethics committees approved all aspects of each survey and all participants gave written informed consent.

### Physical activity

PA was assessed using an established questionnaire that is described elsewhere [21]. Briefly, the interviewer used the questionnaire to enquire about frequency, duration and pace of walking (slow, average, brisk or fast) and participation in sports and exercises using a prompt card showing 10 main groupings, including cycling, swimming, running, football, rugby, tennis and squash. Six open entries could also be recorded. For each sport and exercise, the respondent was asked to specify frequency, duration and perceived intensity. The validity [22] and reliability [23] of the PA questionnaire are described elsewhere. In 2175 adults, the Spearman’s correlation coefficient for accelerometer assessment and questionnaire assessment of moderate- and vigorous-intensity PA was 0.38 (95% CI: 0.32, 0.45) in men and 0.40 (0.36, 0.48) in women [22]. A compendium [24] was used to identify moderate- and vigorous-intensity PA in the present study: moderate activities were of 3.0–5.9 metabolic equivalents (METs) and vigorous activities were of ≥6.0 METs, where one MET is considered to represent resting energy expenditure. Occupational and routine domestic activities were not included in the present analysis. Participants meeting the PA guidelines were defined as reporting at least 150 min per week in moderate-intensity physical activity or at least 75 min per week in vigorous-intensity PA. Inactive’ was defined as not reporting any moderate- or vigorous-intensity physical activities; ‘Insufficiently active’ was defined as reporting physical activity totaling <150 min·wk.-1 in moderate- and <75 min·wk.-1 in vigorous-intensity activities; ‘Weekend warrior’ was defined as reporting ≥150 min·wk.-1 in moderate- or ≥75 min·wk.-1 in vigorous-intensity activities from one or two sessions; ‘Regularly active’ was defined as reporting ≥150 min·wk.-1 in moderate- or ≥75 min·wk.-1 in vigorous-intensity activities from three or more sessions.

### Psychological distress

Common mental disorder was assessed using the 12 item version of the General Health Questionnaire (GHQ-12), a widely-utilized measure of psychological distress in population-based studies [25, 26]. The questionnaire enquires about “How have you been feeling in general over the past few weeks”, for example, “Have you recently been feeling unhappy and depressed?” Participants are required to answer using a likert scale (“not at all”; “no more than usual”; “rather more than usual”; “much more than usual”) scoring one point if symptoms are present, providing total scores in the range of 0–12. We employed a GHQ-12 cut off score of >3 to denote psychological distress, as this threshold has demonstrated the highest Youden index values when depression during the past 2 weeks was the criterion (Y = 0.67, sensitivity 0.84, specificity = 0.84) using Composite International Diagnostic Interview [27].

### Covariates

Trained interviewers asked about age, sex, smoking habit, longstanding illness, and social occupational class. Participants were asked whether they had ‘any longstanding illness, disability or infirmity’, recorded as a binary response (yes/no). Socioeconomic status was determined from participants’ occupations using the four-group version of the Registrar General’s classification: professional and managerial occupations; skilled, non-manual occupations; skilled manual occupations; and, routine and manual occupations. The trained interviewers also measured height and weight that was used to derive body mass index (BMI), expressed as kilograms per meter squared. Obesity was defined as BMI ≥30 kg·m^−2^ in the present study.

### Statistical analysis

Binary logistic regression models were used to estimate the association between physical activity pattern and psychological distress using data pooled across all survey years. Analyses were adjusted for age and sex (Model 1) and further adjusted for smoking habit, longstanding illness, social occupational class, BMI, and survey year (Model 2). We examined effect modification by sex, age, longstanding illness, smoking and obesity. All analyses were performed using SPSS version 22 (IBM Inc.).

## Results

The sample comprised 108,011 participants (age = 47 ± 17 yrs., 46.5% men). Psychological distress (GHQ-12 > 3) was prevalent in 14.5% of the sample. Psychological distress was more prevalent in women, smokers, lower occupational classes and those with chronic illness (Table 1). In models mutually adjusted for all covariates, psychological distress was associated with age (Odds ratio [OR] per year = 0.98, 95% CI, 0.98, 0.99), smoking (OR = 1.46; 1.40, 1.52), female sex (OR = 1.42; 1.37, 1.48), and longstanding illness (OR = 2.65; 2.55, 2.75). In addition, compared to normal BMI (18.5–24.99 kg/m^2^) the odds of psychological distress were higher in underweight (OR = 1.22; 1.03, 1.44) but lower in overweight (OR = 0.92; 0.88, 0.95), and null in the obese (OR = 1.00; 0.95, 1.05).

**Table 1:** Descriptive characteristics in relation to psychological distress (*N* = 108,011)

Variable	GHQ-12 score 0–3 (*N* = 92,382)	GHQ-12 score > 3 (*N* = 15,625)
Age	46.9 ± 17.2	45.8 ± 16.6
Sex (% male)	47.8	38.8
Country (% England)	81.5	79.2
Smoking (%)		
Never	49.3	42.2
Ex-smoker	25.2	22.5
Current	25.5	35.3
Social occupational group		
Professional	5.1	3.6
Managerial	27.9	25.6
Skilled (non-manual/ manual)	43.3	42.5
Semi-skilled manual	17.3	20.4
Unskilled manual	5.9	7.4
Obesity (%)	20.9	23.1
Chronic illness	39.3	60.6
Physical activity		
None	49.3	58.1
Insufficiently active	23.8	22.1
Meets guidelines^a^	26.9	19.8

^a^150 min/wk. of moderate PA, or 75 min/wk. of vigorous PA, or a combination of the two

The regularly active reported on average (SD) 6.6 ± 3.9 sessions MVPA per week compared with weekend warriors who reported 1.1 ± 0.5 sessions/week. Participants with psychological distress were less likely to meet the PA guidelines. There was a dose-response association between PA and psychological distress in models adjusted for all covariates (Table 2). The strongest association was observed in participants meeting the PA guidelines (Table 2); compared with an inactive or an insufficiently active reference group there was 32 and 14% reduced odds of distress, respectively. We also observed lower odds of psychological distress in participants that were active but did not meet the PA guideline (insufficiently active) compared with an inactive reference group, regardless of PA frequency (Table 2). Sub-clinical levels of psychological distress (GHQ-12 score = 1–3) were reported in 25.5% of the sample, thus we performed multinomial regression models to further explore associations with PA. There were inverse associations between all PA patterns and sub-clinical distress, although weaker than those observed with psychological distress defined as GHQ-12 > 3 (Additional file 1: Table S1).

**Table 2:** Associations between physical activity pattern and psychological distress (GHQ-12 > 3). *N* = 108,011

Physical activity pattern	Cases/N	Model 1 Odds Ratio (95% CI)	Model 2 Odds Ratio (95% CI)	Model 3 Odds Ratio (95% CI)
Inactive	9096/54,655	1.0 (Reference)	1.0 (Reference)	
Insufficiently active^a^	2768/20,234	0.73 (0.69, 0.76)	0.81 (0.77, 0.85)	1.0 (Reference)
Insufficiently active^b^	688/5225	0.70 (0.65, 0.76)	0.79 (0.72, 0.86)	1.00 (0.91, 1.10)
Weekend warrior^c^	1001/8921	0.58 (0.54, 0.63)	0.68 (0.63, 0.73)	0.84 (0.77, 0.91)
Regularly active^d^	2100/18,976	0.58 (0.55, 0.61)	0.68 (0.64, 0.72)	0.86 (0.80, 0.92)

Model 1 adjusted for age and sex

Model 2 adjusted for age, sex, smoking, social-occupational class, BMI, longstanding illness, survey year

Model 3 uses covariates from model 2 but insufficiently active as reference category (*N* = 53,356)

Physical activity patterns were defined as follows:

‘Inactive’ was defined as not reporting any moderate- or vigorous-intensity physical activities;

^a^Insufficiently active’ was defined as reporting one or two physical activity sessions per week totaling <150 min·wk.^−1^ in moderate- and <75 min·wk.^−1^ in vigorous-intensity activities

^b^‘Insufficiently active’ was defined as reporting three or more physical activity sessions per week totaling <150 min·wk.^−1^ in moderate- and <75 min·wk.^−1^ in vigorous-intensity activities

^c^‘Weekend warrior’ was defined as reporting ≥150 min·wk.^−1^ in moderate- or ≥75 min·wk.^−1^ in vigorous-intensity activities from one or two sessions

^d^‘Regularly active’ was defined as reporting ≥150 min·wk.^−1^ in moderate- or ≥75 min·wk.^−1^ in vigorous-intensity activities from three or more sessions

The pattern of results was essentially the same in men and women (Additional file 1: Table S2) and across different age categories (Additional file 1: Table S3), although slightly stronger associations were observed in participants >60 yrs. of age (e.g., meeting PA guideline, OR = 0.42; 0.34, 0.52). We observed significant interaction (*p* < 0.05) by longstanding illness (Additional file 1: Table S4), suggesting that in healthy participants the threshold for benefit was observed only at the higher dose of activity (i.e., recommended PA guideline), although associations were evident at lower doses (one or two sessions of MVPA a week under 150 min/wk. threshold) in participants with chronic health conditions. No interactions were observed for smoking (Additional file 1: Table S5) or obesity (Additional file 1: Table S6).

We explored if further benefits could be gained by accumulating PA in certain patterns in participants already meeting the PA guideline. Among those meeting the PA guideline there were no additional benefits observed based on whether PA was accumulated in one or two bouts per week rather than more frequent weekly bouts (Table 2), nor were any associations observed for total volume (Table 3). Compared to participants who reported meeting PA guidelines by taking part in only moderate-intensity PA, women who undertook weekly vigorous intensity PA as part of the guidelines gained additional benefit (three or more weekly sessions, OR = 0.87;0.75, 1.00) (p-trend = 0.006) (Table 4) although this was not observed in men.

**Table 3:** Associations between moderate to vigorous physical activity volume and psychological distress in adults meeting physical activity guidelines (*N* = 27,897)^a^

MVPA volume (min/wk)	Cases/N	Odds Ratio (95% CI)^b^
75–165	791/6743	1.0 (Reference)
166–247	802/6806	1.01 (0.90, 1.12)
248–419	717/6524	0.94 (0.84, 1.05)
>419	802/7535	0.92 (0.82, 1.02)

^a^Here, meeting physical activity guidelines was defined as reporting ≥150 min·wk.^−1^ in moderate-intensity activity, ≥75 min·wk.^−1^ in vigorous-intensity activity, or equivalent combinations

^b^Models adjusted for age, sex, smoking, social-occupational class, BMI, longstanding illness, survey year

**Table 4:** Associations between vigorous-intensity physical activity frequency and psychological distress in adults meeting physical activity guidelines (*N* = 27,897)

Vigorous-intensity physical activity sessions per week	Whole sample Odds Ratio^a^ (95% CI)	Men (*n* = 15,044) Odds Ratio^a^ (95% CI)	Women (*n* = 12,853) Odds Ratio^a^ (95% CI)
None^b^	1.0 (Reference)	1.0 (Reference)	1.0 (Reference)
1–3	1.04 (0.94, 1.14)	1.10 (0.94, 1.29)	1.02 (0.90, 1.15)
> 3	0.93 (0.83, 1.04)	1.03 (0.87, 1.22)	0.87 (0.75, 1.00)

^a^Models adjusted for age, smoking, social-occupational class, BMI, longstanding illness, survey year (and sex in analyses of whole sample)

^b^Here, the reference group is defined as those who reported meeting physical activity guidelines by taking part in ≥150 min·wk.^−1^ of moderate-intensity activity (but no vigorous PA)

## Discussion

The main aim of this paper was to explore the dose-response association between PA and mental health, in particular the ‘weekend warriors’ who do all their exercise in one or two sessions per week. An inverse association between PA and psychological distress was optimal at the recommended PA guideline regardless of whether it was accumulated in 1–2 bouts per week or as more frequent daily bouts. Our results suggest that presence of chronic illness is an important factor in modifying associations between PA and mental health; among participants reporting longstanding health conditions we observed reduced odds of psychological distress below the PA guidelines from as little as one to two sessions per week of MVPA. Given that just under half (~44%) of this general population sample of adults reported a longstanding health condition, this is an important factor in potentially modifying associations between PA and mental health.

Existing studies on dose-response associations have reported an inconsistent pattern of results. Participating in ‘less than’ or ‘greater than’ 150 min MVPA per week has been associated with a variety of effect sizes, ranging 8–63 and 19–27% decreased risk of future depression [8–11], respectively. Other cohort studies [13, 14] have revealed that being active once or twice per week or more than once per week was associated with a decreased risk of depression of up to 40%. The current study is the first to explore if further benefits could be gained by accumulating PA in certain patterns in participants already meeting the PA guideline. An analysis of over 600,000 adults of all ages from the US and Europe, showed that a nearly optimal threshold for longevity occurred at 3 to 5 times the PA recommendation (39% reduction in all-cause mortality) although the additional benefit over and above doses corresponding to 1–2 multiples of the PA guideline (31% reduction in all-cause mortality) was rather modest [28]. Our results suggested that there were no additional mental health benefits over and above the PA guidelines (see Table 3). The addition of weekly vigorous intensity PA, however, could provide additional benefit, particularly in women. Data from randomized controlled trials have tended to show that light/moderate intensity exercise has greater anti-depressive effects [29], effects on positive mood [30], and on reducing symptoms of fatigue compared with vigorous intensity [31, 32]. Nevertheless, these effects may be modified by physical fitness in that fitter people training more regularly could gain from more vigorous exercise. Moreover, studies of acute bouts of exercise suggest that affective responses become more negative as the intensity increases, yet rebound back to more positive affective states after exercise. There are also strong intra-individual variations in affective responses to exercise at levels below highly vigorous [33]. In addition, more vigorous physical activity may be too challenging for some, and is associated with higher dropout, thus reducing the chances of gaining mental health benefit [34].

The main limitation of this study is the cross-sectional design, which precludes us from making any inferences about direction or causality. Reverse causation is possible in that people with poor mental health may find it difficult to be physically active. Nevertheless, hospitalized patients with severe mental health problems would have been excluded from our study as only community dwelling participants were surveyed. In addition, we also found associations between PA and sub-clinical levels of distress, which is less likely to be driven by reverse causation. Our PA measures were designed to capture moderate to vigorous intensity PA and thus we could not examine other parts of the PA spectrum such as light intensity and sedentary behaviours. Indeed sedentary behaviour has been longitudinally associated with risk of future depression in some studies [35]. Undoubtedly, controlled trials are the best test of causality. However, studies of community samples have advantages in that they are more representative. In the present study we aimed to minimise possible confounding by controlling for key covariables.

## Conclusion

In conclusion, these data show that mental health benefits may be accrued through different patterns of physical activity. Personalized medicine approaches tailored to individual preferences and time availability during the week should therefore be encouraged when prescribing exercise.

## Additional file

Additional file 1: Supplementary analyses. (DOCX 24 kb)
