# Supplementary material for: Weekend warrior physical activity pattern and common mental disorder: a population wide study of 108,011 British adults
Source: Int J Behav Nutr Phys Act. 2017 Jul 14;14:96. doi: 10.1186/s12966-017-0549-0 (PMC5513116; doi:10.1186/s12966-017-0549-0)
Supplement: Additional file 1: — Supplementary analyses. (DOCX 24 kb) [file 12966_2017_549_MOESM1_ESM.docx]

Additional file 1: **Supplementary analyses**

**Table S1.** Multinomial regression to examine association between physical activity pattern and psychological distress

| **PA category** | Sub-clinical psychological distress  (GHQ-12 = 1 – 3) | Psychological distress  (GHQ-12 >3) |
| --- | --- | --- |
| Inactive | 1.0 (Ref) | 1.0 (Ref) |
| Insufficiently active^1^ | 0.92 (0.88, 0.96) | 0.78 (0.74, 0.82) |
| Insufficiently active^2^ | 0.91 (0.85, 0.98) | 0.76 (0.70, 0.83) |
| Weekend warrior^3^ | 0.88 (0.83, 0.93) | 0.64 (0.59, 0.69) |
| Regularly active^4^ | 0.79 (0.76, 0.83) | 0.62 (0.59, 0.66) |

The reference category for dependent variable is GHQ-12 = 0.

Odds ratio (OR) adjusted for age, sex, smoking, social-occupational class, BMI, longstanding illness, survey year.

‘Inactive’ was defined as not reporting any moderate- or vigorous-intensity physical activities; ^1’^Insufficiently active’ was defined as reporting one or two physical activity sessions per week totaling <150 min·wk^-1^ in moderate- and <75 min·wk^-1^ in vigorous-intensity activities;

^2^‘Insufficiently active’ was defined as reporting three or more physical activity sessions per week totaling <150 min·wk^-1^ in moderate- and <75 min·wk^-1^ in vigorous-intensity activities;

^3^‘Weekend warrior’ was defined as reporting ≥150 min·wk^-1^ in moderate- or ≥75 min·wk^-1^ in vigorous-intensity activities from one or two sessions;

^4^‘Regularly active’ was defined as reporting ≥150 min·wk^-1^ in moderate- or ≥75 min·wk^-1^ in vigorous-intensity activities from three or more sessions .

**Table S2**. Associations between physical activity pattern and psychological distress stratified by sex.

| Physical activity pattern | **Men**  (n=50,243)  OR (95% CI) | **Women** (n=57,768)  OR (95% CI) |
| --- | --- | --- |
| Inactive | 1.0 (Reference) | 1.0 (Reference) |
| Insufficiently active^1^ | 0.81 (0.75, 0.88) | 0.81 (0.75, 0.85) |
| Insufficiently active^2^ | 0.75 (0.65, 0.87) | 0.82 (0.73, 0.91) |
| Weekend warrior ^3^ | 0.66 (0.59, 0.74) | 0.71 (0.64, 0.78) |
| Regularly active^4^ | 0.62 (0.57, 0.68) | 0.74 (0.69, 0.80) |

Odds ratio (OR) adjusted for age, smoking, social-occupational class, BMI, longstanding illness, survey year.

**Table S3**. Associations between physical activity pattern and psychological distress stratified by age.

| Physical activity pattern | **18 – 40 yrs**  (n=42,259)  OR (95% CI) | **41 – 60 yrs**  (n=38,494)  OR (95% CI) | **> 60 yrs**  (n=25,601)  OR (95% CI) |
| --- | --- | --- | --- |
| Inactive | 1.0 (Reference) | 1.0 (Reference) | 1.0 (Reference) |
| Insufficiently active^1^ | 0.88 (0.82, 0.95) | 0.80 (0.74, 0.86) | 0.64 (0.55, 0.74) |
| Insufficiently active^2^ | 0.94 (0.82, 1.06) | 0.75 (0.65, 0.86) | 0.52 (0.38, 0.71) |
| Weekend warrior ^3^ | 0.75 (0.68, 0.83) | 0.66 (0.58, 0.75) | 0.47 (0.35, 0.64) |
| Regularly active^4^ | 0.82 (0.76, 0.89) | 0.63 (0.57, 0.70) | 0.42 (0.34, 0.52) |

Odds ratio (OR) adjusted for age, sex, smoking, social-occupational class, BMI, longstanding illness, survey year.

**Table S4**. Associations between physical activity pattern and psychological distress stratified by longstanding illness.

| Physical activity pattern | **Healthy**  (n=62,263)  OR (95% CI) | **Unhealthy** (n=45,748)  OR (95% CI) |
| --- | --- | --- |
| Inactive | 1.0 (Reference) | 1.0 (Reference) |
| Insufficiently active^1^ | 0.95 (0.88, 1.02) | 0.72 (0.68, 0.77) |
| Insufficiently active^2^ | 1.09 (0.97, 1.23) | 0.59 (0.52, 0.67) |
| Weekend warrior ^3^ | 0.83 (0.75, 0.92) | 0.58 (0.52, 0.64) |
| Regularly active^4^ | 0.87 (0.80, 0.94) | 0.54 (0.50, 0.59) |

Odds ratio (OR) adjusted for age, sex, smoking, social-occupational class, BMI, survey year.

**Table S5**. Associations between physical activity pattern and psychological distress stratified by smoking history.

| Physical activity pattern | **Never**  (n=52,132)  OR (95% CI) | **Ex-smoker**  (n=26,773)  OR (95% CI) | **Current**  (n=29,106)  OR (95% CI) |
| --- | --- | --- | --- |
| Inactive | 1.0 (Reference) | 1.0 (Reference) | 1.0 (Reference) |
| Insufficiently active^1^ | 0.78 (0.73, 0.84) | 0.71 (0.64, 0.79) | 0.85 (0.78, 0.92) |
| Insufficiently active^2^ | 0.71 (0.63, 0.80) | 0.62 (0.51, 0.74) | 0.88 (0.78, 1.01) |
| Weekend warrior ^3^ | 0.69 (0.60, 0.78) | 0.60 (0.50, 0.74) | 0.73 (0.62, 0.86) |
| Regularly active^4^ | 0.66 (0.60, 0.72) | 0.60 (0.53, 0.68) | 0.78 (0.71, 0.86) |

Odds ratio (OR) adjusted for age, sex, social-occupational class, BMI, longstanding illness, survey year.

**Table S6**. Associations between physical activity pattern and psychological distress stratified by obesity.

| Physical activity pattern | **Non-obese**  (n=85,072)  OR (95% CI) | **Obese**  (n=22,939)  OR (95% CI) |
| --- | --- | --- |
| Inactive | 1.0 (Reference) | 1.0 (Reference) |
| Insufficiently active^1^ | 0.80 (0.76, 0.84) | 0.76 (0.68, 0.84) |
| Insufficiently active^2^ | 0.75 (0.69, 0.82) | 0.73 (0.61, 0.88) |
| Weekend warrior ^3^ | 0.69 (0.62, 0.76) | 0.68 (0.55, 0.84) |
| Regularly active^4^ | 0.71 (0.67, 0.76) | 0.57 (0.49, 0.67) |

Odds ratio (OR) adjusted for age, sex, smoking, social-occupational class, longstanding illness, survey year.
